# Emerging Mesoporous Polyacrylamide/Gelatin–Iron Lanthanum Oxide Nanohybrids towards the Antibiotic Drugs Removal from the Wastewater

**DOI:** 10.3390/nano13212835

**Published:** 2023-10-26

**Authors:** Nazish Parveen, Fatimah Othman Alqahtani, Ghayah M. Alsulaim, Shada A. Alsharif, Kholoud M. Alnahdi, Hasna Abdullah Alali, Mohamad M. Ahmad, Sajid Ali Ansari

**Affiliations:** 1 Department of Chemistry, College of Science, King Faisal University, Al-Ahsa, P.O. Box 380, Hofuf 31982, Saudi Arabia; foqahtani@kfu.edu.sa (F.O.A.); g.m.alsulaim@gmail.com (G.M.A.); 2 University College of Umlij, University of Tabuk, Tabuk 71491, Saudi Arabia; shalsharif@ut.edu.sa; 3 Department of Physics, Faculty of Science, University of Tabuk, Tabuk 71491, Saudi Arabia; alnahdi.kholoud@gmail.com; 4 Department of Physics, College of Science, King Faisal University, Al-Ahsa, P.O. Box 400, Hofuf 31982, Saudi Arabia; hasali@kfu.edu.sa (H.A.A.); mmohamad@kfu.edu.sa (M.M.A.); 5 Department of Physics, Faculty of Science, The New Valley University, El-Kharga 72511, Egypt

**Keywords:** adsorption, nanohybrids, diclofenac, gelatin, kinetic models

## Abstract

The polyacrylamide/gelatin–iron lanthanum oxide (P-G-ILO nanohybrid) was fabricated by the free radical grafting co-polymerization technique in the presence of N,N-methylenebisacrylamide (MBA) as cross linker and ammonium persulfate (APS) as initiator. The P-G-ILO nanohybrid was characterized by the various spectroscopic and microscopic techniques that provided the information regarding the crystalline behavior, surface area, and pore size. The response surface methodology was utilized for the statistical observation of diclofenac (DF) adsorption from the wastewater. The adsorption capacity (*q*_e_, mg/g) of P-G-ILO nanohybrid was higher (254, 256, and 258 mg/g) than the ILO nanoparticle (239, 234, and 233 mg/g). The Freundlich isotherm model was the best fitted, as it gives the higher values of correlation coefficient (R^2^ = 0.982, 0.991 and 0.981) and lower value of standard error of estimate (*SEE* = 6.30, 4.42 and 6.52), which suggested the multilayered adsorption of DF over the designed P-G-ILO nanohybrid and followed the pseudo second order kinetic model (PSO kinetic model) adsorption. The thermodynamic study reveals that adsorption was spontaneous and endothermic in nature and randomness onto the P-G-ILO nanohybrids surface increases after the DF adsorption. The mechanism of adsorption of DF demonstrated that the adsorption was mainly due to the electrostatic interaction, hydrogen bonding, and dipole interaction. P-G-ILO nanohybrid was reusable for up to five adsorption/desorption cycles.

## 1. Introduction

The dumping of aqueous as well as solid wastes adjacent to the water bodies around the world has pushed towards an approaching worldwide water calamity. The water quality of the natural resources, and consequently the drinking water supply, is one of the major issues of our industrialized civilization [1]. The growth of the various industries such as chemical, pharmaceutical, petrochemical, and agrifood generates large amounts of waste materials. A number of organic pollutants such as organic dyes, pesticides, phenols, and hydrocarbons have been noticed in the worldwide environment in recent periods [2]. However, pharmaceutical drugs are known as an emerging class of pollutants, despite their presence in water for decades. Different types of pharmaceutical compounds such as antibiotics, antidiabetics, analgesics, antiseptics, and antimicrobials have been frequently used as important drugs. The pharmaceutical micropollutants mainly enter into the environment through the raw wastewater and the effluents released from conventional wastewater treatment plants [3]. Theses pharmaceutical wastes are mostly not degraded completely after application. Subsequently, these compounds enter into the ecosystem, which is responsible for the environmental risk and human health problems [4]. 

Diclofenac (DF) is a nonsteroidal anti-inflammatory drug (NSAID) and has been accounted as nearly one of the most extensively produced (940 tons/year) worldwide [5]. Diclofenac is mostly used to treat pain, problem with joints, muscles and bones, dry, scaly patches of skin caused by sun damage, etc. Therefore, landfilling of these wastes is an invitation for groundwater pollution [6]. Recently, attention has been placed on the sequestration of micro pollutants from the wastewater due to their aquatic pollution, resistance to pathogens, and genotoxicity and endocrine disruption [7]. The toxicological appearances of these drugs have stimulated the development of numerous techniques for aquatic pollutants remediation, in which adsorption is known as the best technique because of its simplicity and low operational cost, saving time along with reducing the possibility of other pollutants and toxic sludge formation [8]. Polymer-based materials were used as potential materials for the sequestration of micro pollutants, owing to the surface chemistry modification, high surface area, and surface regeneration under mild condition [9]. The polymeric materials such as polymer hydrogels and polymer hydrogel nanocomposite have been found as propitious adsorbents. The high adsorption efficiency of polymeric materials has been ascribed to their several functional groups (hydroxyl, amino, amide, and carboxyl), swelling properties, and three-dimension cross-linked polymeric design [10]. However, the main drawback of polymeric materials is their weak mechanical strength, which restricts their usage as adsorbents. The mechanical strength of these polymeric materials can be enhanced by the implantation of several materials such as nano metal oxides, zeolite, and nano clays [11]. Lately, iron-based metal oxide has been proven as an excellent material for wastewater treatment. FeLaO_3_ (ILO) is a perovskite oxide with the general formula ABO_3_. IFO has garnered interest because of its remarkable magnetic and dielectric properties, and it also increases the mechanical strength of composite materials. The literature review suggested that researchers have been synthesizing the polymer-based materials for removing various pollutants including metal, dyes, and drugs. The TiO_2_-chitosan composite [12], TiO_2_-hydrogel nanocomposite [13], magnetic amine-functionalized chitosan for diclofenac sodium [14], sodium alginate-TiO_2_ hydrogel nanocomposite [15], polyacrylamide-g-chitosan γ-Fe_2_O_3_ nanocomposite for malachite green [16], and magnetic-based nanocomposite [17,18] have been used for removal of dyes and metals ions from the wastewater. 

In this study, the polyacrylamide/Gelatin-FeLaO_3_ nanocomposite (P-G-ILO nanohybrid) adsorbent was fabricated. Herein, the point of novelty is the synthesis of P-G-ILO nanohybrid via the free radical grafting copolymerization technique in the presence of N,N-methylenebisacrylamide (MBA) as cross linker and ammonium persulfate (APS) as initiator for the confiscation of diclofenac from the aqueous solution. Meanwhile, the ILO nanoparticles were for the first time incorporated into the polyacrylamide/gelatin matrix. To our best knowledge, no study on the synthesis and characterization of P-G-ILO nanohybrid has been reported so far in the literature. 

The optimization of adsorption parameters was evaluated by the statistical method using response surface methodology (RSM), which is the operative statistical procedure for the evaluation of optimum conditions for adsorption studies because it reduces the number of experiments and provides the interaction of the adsorption parameters. The nonlinear isotherm (Langmuir and Freundlich), pseudo first order kinetic models (PFO kinetic models), and pseudo second order kinetic models (PSO kinetic models) were used to investigate the DF adsorption capacity and rate of the DF adsorption. The adsorption capacity of the ILO was compared with the P-G-ILO nanohybrids. The mechanism of the adsorption suggested that DF was mainly adsorbed onto the P-G-ILO nanohybrid surface through electrostatic interaction, however, there was also the possibility of hydrogen bonding and dipole interaction. The cyclic stability and reusability of the P-G-ILO nanohybrid was also studied using 0.1 M NaOH solution.

## 2. Materials and Methods

Analytical grade (AG) chemicals were used for the synthesis of the P-G-ILO nanohybrid. The salts such as lanthanum nitrate (99%), ferrous nitrate (99%), and sodium chloride (99.5) were acquired from Merck Saudi Arabia (Riyadh, Saudi Arabia). Acrylamide (99.5%) was bought from Spectrochem (Bangalore, India). Gelatin and MBA (99%) were acquired from CDH (Atlanta, GA, USA). Ammonium hydroxide, sodium hydroxide (98%), and hydrogen chloride (37%) were purchased from Merck Saudi Arabia. The diclofenac sodium (98%) was obtained from Merck Saudi Arabia.

### 2.1. Synthesis of Iron Lanthanum Oxide Nanoparticles

The iron lanthanum oxide (FeLaO_3_) was synthesized by the co-precipitation method. The separate solutions of 0.1 M ferrous nitrate and Lanthanum nitrate were prepared separately in deionized water. The three solutions were mixed under mechanical magnetic stirring for 40 min at 60 °C. Afterwards, NH_4_OH solution was added dropwise to increase the pH of the above mixture up to pH 10 with constant heating and stirring. After 1 h, the precipitate of iron lanthanum oxide was obtained and further washed with water and dried at 70 °C. The dried sample was further calcined at 500 °C for 4 h. 

### 2.2. Synthesis of P-G-ILO Nanohybrid

The P-G-ILO nanohybrid was produced by copolymerization of acrylamide onto the gelatin surface through the free radical grafting co-polymerization technique in the presence of FeLaO_3_ nanoparticle and APS as initiator. Separate aqueous solutions (100 mL) of gelatin and acrylamide (2% *w*/*w*) were prepared. Both solutions were mixed under continuous stirring, to which different known amounts (0.25 g, 0.5 g, 0.75 g) of ILO nanoparticle were added. The initiation reaction was started by adding the 0.25 M solution of APS initiator. After 10 min, the MBA cross-linker was added, and stirring was continued for 4 h at 70 °C. The mixture was cooled, and the nanocomposite thus obtained was washed using ethanol and deionized water. The P-G-ILO nanohybrid was dried in hot air for 24 h. The nanohybrid with 0.5 g of ILO nanoparticle shows better adsorption behaviors, hence was chosen for the further studies. 

### 2.3. Characterization

The structural characterization of P-G-ILO nanohybrid was done by FTIR spectroscopy (BX-spectrum, PerkinElmer, Waltham, MA, USA) and X-ray diffraction analysis (Analytica PW1830, Mumbai, India). The BET surface area analyzer (Nova 2200e QuantaChrome, Boynton Beach, FL, USA) investigated the specific surface area of ILO and P-G-ILO nanohybrids. The surface and internal structure of the P-G-ILO nanohybrid was determined by transmission electron microscope (Technai, F30 S-Twin, Pozzolo, Italy) and scanning electron microscope (Zeiss, Jena, Germany, SIGMA VP). 

### 2.4. Design of Experiments for the Adsorption of DF

The experiments were constructed using RSM combined with a central composite designing system (CCD) to determine the optimum adsorption of DF onto the P-G-ILO nanohybrid nanocomposite surface. The experimental design consisted of four factors that were estimated at three levels: −1, 0, +1, where −1, 0, +1 are the low, central and high values, respectively (Table 1). Four experimental parameters: A—concentration (mg/L), B—pH, C—time (min), and D—dose (g/L) were picked as independent variables influencing the separation of DF from the aqueous solutions. The correlation coefficient and quadratic model equation were produced through modeled software and authenticated by observed experimental data. The adsorption capacity (*q*_e,_ mg/g) of the DF was taken to be the response of system studies.

The response (adsorption capacity of P-G-ILO nanohybrid) obtained from the experimental runs was fitted to a quadratic polynomial equation (Equation (1)) to determine the correlation between the linear, quadric, and interaction effects in terms of the code factors.

(1) $$y^{N}=\beta_{0}+\overset{k}{\underset{i=1}{\sum }}\beta_{i}x_{i}+\overset{k}{\underset{i=1}{\sum }}\beta_{ii}x_{i}^{2}+\overset{k}{\underset{i=1}{\sum }}\overset{k}{\underset{j=1}{\sum }}\beta_{ij}x_{i}x_{j}+\alpha_{i\neq j}$$

where *y^N^* is the response (adsorption capacity), k represents the number of variables, *β*_0_ is the coefficient constant, and *β_i_*, *β_ii_*, and *β_ij_* are coefficients of the linear, quadratic, and interaction effects, whereas *x_i_* and *x_j_* represent the coded independent variables, and α is the error.

### 2.5. Isotherm Kinetic and Thermodynamic Study 

The proficiency, adsorption rate, feasibility, and mechanism of adsorption of DF onto P-G-ILO nanohybrid surface was achieved by isotherm kinetic and thermodynamic studies. The isotherm studies were performed by shaking 25 mL solution of varying concentrations (50–100 mg/L) of DF into 50 mL conical flasks at pH 6 for 45 min with 0.4 g/L P-G-ILO nanohybrid dose. The reaction was carried out at the three different (298 K, 303 K, and 308 K) temperatures in a water shaker bath. However, kinetic experiments were executed by shaking 25 mL DF solution (90, 100 mg/L) of pH 6 with 0.4g/L P-G-ILO nanohybrid dose for a 10–60 min time interval at 308 K. The adsorbent was separated by centrifugation, and the residual amount of DF was determined by a UV visible spectrophotometer. The following relationships were used to calculate the amount of DF absorbed at equilibrium (mg/g), *q*_e_ (Equation (2)), and time *t*, *q*_t_ (Equation (3)):

(2) $$\;q_{e}=\left[\frac{C_{o}-C_{e}}{W_{NC}}\times V\right]$$

(3) $$q_{t}=\left[\frac{\left(C_{o}-C_{t}\right)}{W_{NC}}\times V\right]$$

where *C_o_*, *C_e_*, and *C_t_* are initial DF concentration, equilibrium DF concentration, and DF concentration at any time *t*, respectively (mg/L). *W_NC_* is the mass (g) of the P-G-ILO nanohybrid. 

The thermodynamic study was carried out by equilibrating the DF solution (80, 90, 100 mg/L) on optimized conditions at 298 K, 303 K, and 308 K. The thermodynamic study reports the feasibility and spontaneity of the adsorption of DF by the P-G-ILO nanohybrid.

## 3. Result and Discussion

### 3.1. Structural and Morphological Characterization

**Figure 1:**
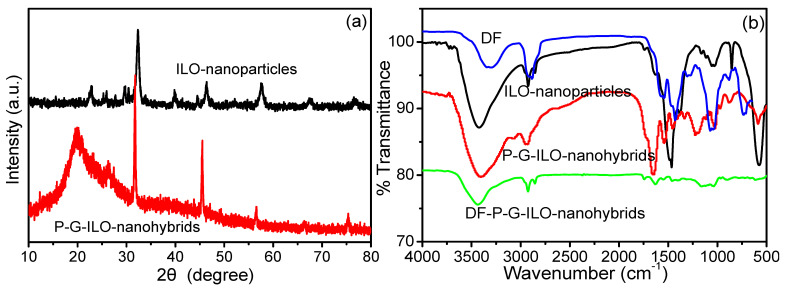
(**a**) XRD pattern of the ILO nanoparticles and P-G-ILO monohybrids and (**b**) FTIR spectra of the ILO nanoparticles, DF, P-G-ILO monohybrids, and DF-G-ILO monohybrids.

The FeLaO_3_ nanoparticles and P-G-ILO nanohybrids were characterized by the X-ray diffraction analysis. The XRD spectrum of ILO shows that the nanoparticles are purely crystalline in nature (Figure 1a). The spectrum of ILO demonstrated intense characteristic peaks at 2Ө° values with diffraction patterns 22.64 (110), 25.90 (111), 32.29 (121), 39.8 (220), 46.33 (202), 57.48 (240), and 67 (242) and are well-matched to the JCPDS number 37-1493 [19]. Scherrer’s equation was used to compute the particle size of the ILO nanoparticles (Equation (4)).

D = Kλ/βcosθ(4)

where D = nano crystallite size, K = shape factor, and β = width of the peak of the diffraction at half-maximum (FWHM). The average particle size of the nanoparticle was calculated to be 12.17 nm. The spectra of P-G-ILO nanohybrid exhibit a broad band from 10–20°, which shows the presence of polymer, and the characteristic peaks of ILO nanoparticle were also detected in nanocomposite with slight shift and reduced intensity (32.29° to 31.76°, 46.33° to 45.48°, 57.48° to 56.47°, and 67° to 66.42°), which might be due to the difference in the ionic radii of the nanoparticles and polymer matrix [20]. The average crystalline size of P-G-ILO nanohybrid was calculated to be 56 nm. 

The FTIR spectra of the DF, ILO nanoparticles, P-G-ILO nanohybrid, and DF-P-G-ILO nanohybrid are depicted in Figure 1b. The FTIR spectra of the ILO show peaks at 3433, 1471, 1032, and 575 cm^−1^. The characteristic peak at 3433 cm^−1^ was identified for the –OH stretching vibration [21]. The intense peak at 575 cm^−1^ was acknowledged for the characteristic band of Fe–O stretching [22]. The spectrum of P-G-ILO nanohybrids exhibits peaks at 3413 and 2923 cm^−1^, assigned for NH/OH stretching and C–H vibrations, respectively [23]. The sharp peaks at 1648, 1538, and 1205 cm^−1^ might be due to the presence of the I, II, and III amide. The I amide band originated due to the –C=O stretching vibration, whereas the II and III amides originated due to the –NH bending vibration and –C-N stretching vibration [24]. The specific bands at 595 and 460 might be due the presence of Fe–O and La–O bonds. However, the FTIR spectrum of DF-loaded P-G-ILO nanohybrids shows a slight shift in the peak, from 3413 cm^−1^ to 3435 cm^−1^, 1648–1619 cm^−1^ and 595–615 cm^−1^, signifying the electrostatic interaction between the positively charged P-G-ILO nanohybrid surface and negatively charged DF molecule. The presence of a new band at 720 cm^−1^ might be accounted for by the C–Cl of DF, which suggested the existence of DF on P-G-ILO nanohybrid [25]. 

**Figure 2:**
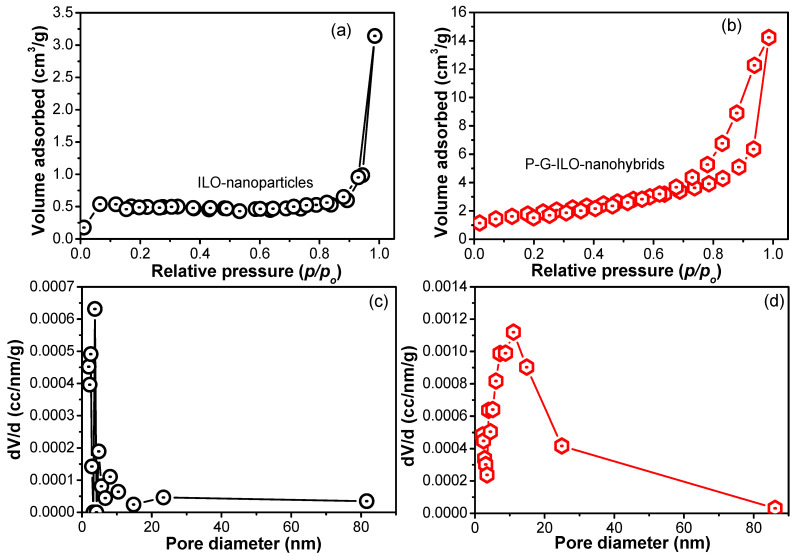
The nitrogen adsorption/desorption graph for (**a**) ILO nanoparticles and (**b**) P-G-ILO nanohybrids, and (**c**) pore diameter profiles of the ILO nanoparticles and (**d**) P-G-ILO nanohybrids.

As the synthesized P-G-ILO nanohybrid is used for the removal of DF from an aqueous environment, the surface area is an important parameter. The BET surface area analyzer investigated the surface area of the synthesized ILO and P-G-ILO nanohybrids. The N_2_ adsorption/desorption curves of the ILO and P-G-ILO nanohybrids are displayed in Figure 2. It was manifested that adsorption isotherm belongs to type IV isotherm, which suggested the mesoporous structure of the nanocomposite [26]. The BET surface area of the ILO nanoparticles was found to be 2.43 m^2^/g, with pore diameter of 3.07 nm and 0.006 cc/g of pore volume. However, after the amalgamation of nanoparticles into the polymer matrix, the surface area became 7.5 m^2^/g with 11.06 nm of pore diameter and 0.023 cc/g of pore volume. This increase in the surface area might be accounted for by the high adsorption efficiency of the P-G-ILO nanohybrids.

**Figure 3:**
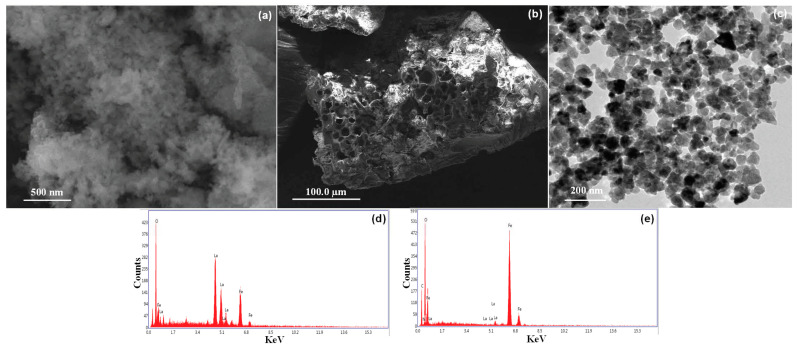
(**a**) SEM image of the ILO nanoparticle, (**b**) SEM image of the P-G-ILO nanohybrid, (**c**) TEM image of the P-G-ILO nanohybrid, (**d**) EDX spectra of the ILO nanoparticle, and (**e**) EDX spectra of the P-G-ILO nanohybrid.

To understand the surface morphology, the ILO and P-G-ILO nanohybrids and DF-P-G-ILO were analyzed by scanning electron microscope. SEM images of ILO and P-G-ILO nanohybrid are specified in Figure 3a,b. The surface of nanocomposite shows that the nanoparticles were arrange in irregular fashion. The surface of the nanoparticle was heterogeneous in nature. However, the P-G-ILO nanohybrid shows continuous pores of different size. After the adsorption of the DF, the surface pores of P-G-ILO nanohybrid was covered by the DF molecule (Figure S1a,b). TEM images determined the internal morphology of the P-G-ILO nanohybrid (Figure 3c). The TEM image of the P-G-ILO nanohybrid demonstrated that the ILO nanoparticles were implanted into the polymer backbone (Figure 3c). The overall particle size of the P-G-ILO nanohybrid was evaluated from the particle size distribution curve (Figure 3c). The average size of the particles was assessed to be 50–55 nm and was in good agreement with the size calculated from XRD patterns.

EDX spectra of ILO display the occurrence of elements such as iron, lanthanum, and oxygen and presence of new elements such as C and nitrogen. In the EDX spectra, P-G-ILO nanohybrids supported the formation of nanocomposite (Figure 3d,e). 

The pHzpc of the P-G-ILO nanohybrid was determined by the batch method. The P-G-ILO nanohybrid (0.5 g) was added into 50 mL of 0.1 M NaCl solution in different conical flasks. Initial pHs (pHi) of P-G-ILO nanohybrids containing solution were adjusted from 2 to 12 by the addition of 0.1 M NaOH/or HCl solution. The solution was equilibrated for 24 h, and final pHs of the solution were measured. The plot of ∆pH vs. pHi (Figure S2) gave the value of pHzpc [27].

The CCD of thirty experiments was prepared to investigate the interaction of four prominent factors (DF concentration, pH, time, and P-G-ILO nanohybrid dose) in the adsorption process. The experimental data were evaluated by multiple regressive CCD analysis, which gave the polynomial quadratic equation, which reflects the relationship between the response and actual input variable (Equation (5))

*q*_e_ = 87.50 + 2.94 × Concentration + 5.58 × pH + 0.272 × Time − 281.628 × Dose − 0.048 × Concentration × pH + 0.0022 × Concentration × Time − 1.50864 × Concentration × Dose + 0.0069 × pH × Time + 4.46 × pH × Dose − 0.063 × Time × Dose − 0.0002 × Concentration2 − 0.79 × pH2 − 0.0056 × Time2 × 142.6 × Dose2 (5)

The acceptability of the quadratic model was authenticated through the analysis of variance (ANOVA). The ANOVA of DF adsorption using P-G-ILO nanohybrid is summarize in the table (Table 2). The model values of the F = 175.46 suggests the applicability of the model. The *p* value of 0.0001 being less than the confidence level (0.05) infers the permissibility of the model. Values of Prob > F (*p*-value) less than (0.05) specify the model in terms of its significance. In this case A, B, D, AB, AD, BD, B^2^, and D^2^ are the significant model terms, indicating that these terms contributed significantly to assist the model.

**Figure 4:**
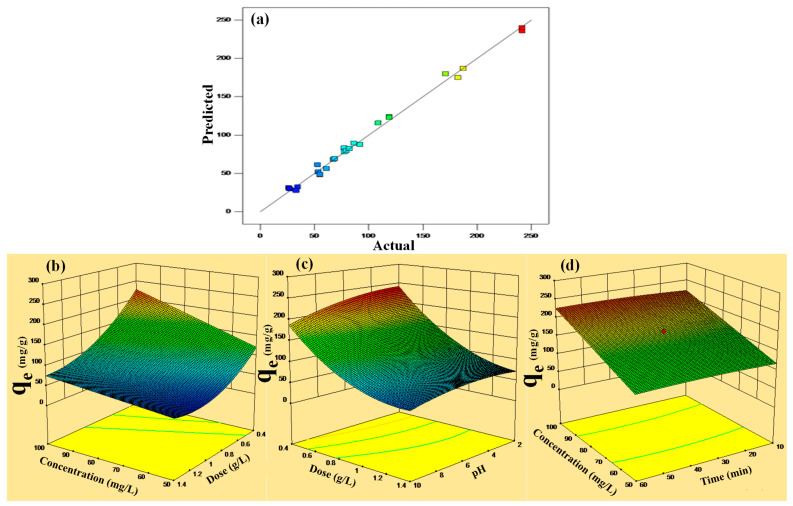
(**a**–**d**) Predicted vs. actual plot of *q*_e_ and 3D plots of the interaction of the independent factors and *q*_e_ at optimized conditions.

In addition, the *R*^2^ value is more near to unity (0.961), and the smaller value of standard deviation (6.16) extremely supported the accuracy of response (adsorption capacity) suggested by the quadratic model. The 0.961 *R*^2^ value suggested that about 96.1% of the changeability could be clarified in random fashion variations in the variable parameter. The “Pred *R*-Squared” of 0.988 is in suitable agreement with the “Adj *R*-Squared” of 0.999, as the difference is less than 0.2 (Table 3). The actual vs. predicted plots (Figure 4a) elucidate that actual and predicated values are displaying a regular distribution along a straight line. The actual and predicted values of the maximum adsorption capacity of P-G-ILO nanohybrid for the DF adsorption are given in the table (Table S1). 

### 3.2. Variable Effects of the Adsorption Capacity of DF

The three-dimensional graph for removal of DF explains the relation of the adsorption capacity of CF and applied different parameters. Figure 4b illustrates the simultaneous impact of concentration and contact time onto the amputation of DF when keeping constant P-G-ILO nanohybrid dose (0.4 g/L) and pH (6). The *q*_e_ values demonstrate an antagonistic effect with change in concentration. The upsurge in *q*_e_ is owed to the large dynamic force made by higher initial DF concentration that diminishes the mass transfer resistance, which boosts the adsorption capacity. 

The combined effect of PAG-ILO nanocomposite dose and DF concentration onto the adsorption capacity was studied by fixing constant other parameters (Figure 4c). It was found that by increasing DF concentration from 50 to 100 (mg/L), the *q*e increases, and by increasing the nanocomposite dose from 0.4 to 1.4 (g/L), the adsorption capacity decreases rapidly; this might be due to less availability of DF molecules in the solution. 

The change in the adsorption capacity due to the shared influence of P-G-ILO nanohybrid dose and DF solution pH is displayed in Figure 4d. As the solution pH increases from 2 to 6, the adsorption capacity increases initially. The *pH*_zpc_ of the adsorbent was 6.3, therefore the P-G-ILO nanohybrid surface was positively charged at pH < *pH*_zpc_, and the *pK*_a_ of DCF molecules is four. Initially, there is less interaction between the positively charged P-G-ILO nanohybrid surface and undissociated DF molecules; after pH 4, the existence of electrostatic interaction between positively charged P-G-ILO nanohybrid and negatively charged DF molecules was found [28]. After further increase in the pH from 6 to 10, the adsorption capacity decreases slightly; this might be due to the repulsion between negatively charged P-G-ILO nanohybrid and negatively charged DF molecules. 

### 3.3. Optimization of DF Adsorption Parameters

The optimum condition for the adsorption of DF from aqueous solution onto the P-G-ILO nanohybrid was evaluated by the numerical optimization studies. The statistical optimization was carried out by using optimization parameters such as DF concentration, P-G-ILO nanohybrid dose, and contact time in range. The solution pH was fixed at 6, and the adsorption capacity (*q*_e_) was defined as maximum to accomplish the highest performance. The optimized values were found to be 100 mg/L DF concentration, 45 min of contact time, 0.4 g/L of P-G-ILO nanohybrid dose, and pH = 6. In other word, the maximum adsorption capacity of 227 mg/g was observed in the case of the P-G-ILO nanohybrid. 

### 3.4. Mechanism of the Adsorption of DF Molecules onto the P-G-ILO Nanohybrid

**Scheme 1:**
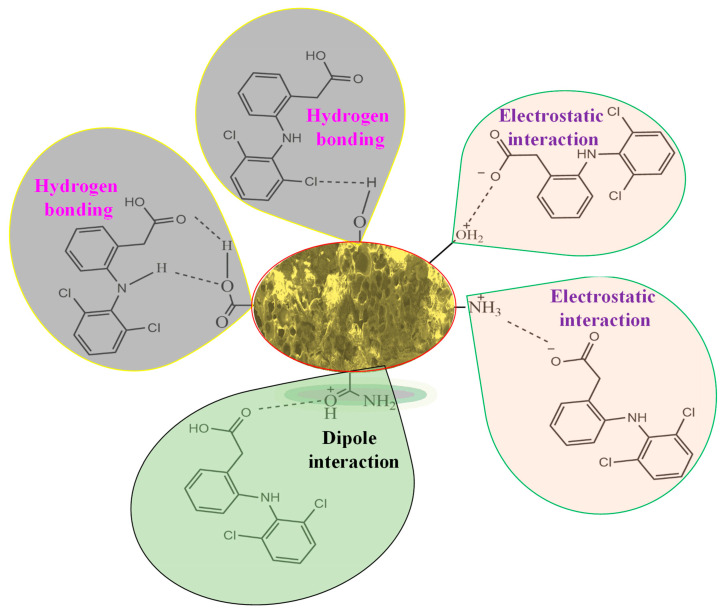
Plausible mechanism of the adsorption of DF onto the P-G-ILO nanohybrid.

The plausible mechanism of adsorption of DF molecules onto the P-G-ILO nanohybrid is specified as Scheme 1. The mechanism is mainly elucidated from the pH studies and FTIR spectra of DF-loaded P-G-ILO nanohybrid. At pH 5–6 (>*pH*_zpc_), the P-G-ILO nanohybrid surface was positively charged, and DF molecules were negatively charged (*pK*_a_ = 4.1), so the electrostatic interaction existed. The existence of the electrostatic interaction as well as hydrogen bonding and dipole interaction were supported by the above-mentioned characterization results. For example, FTIR spectra of the P-G-ILO nanohybrid and DF-loaded P-G-ILO nanohybrid. The shift in characteristic peaks of NH/OH was from 3413 cm^−1^ to 3435 cm^−1^, amide I was from 1648 to 1619 cm^−1^, and Fe–O was from 595 to 615 cm^−1^, which suggested the interaction of various functional groups of the P-G-ILO nanohybrid with the DF molecule. 

### 3.5. Nonlinear Langmuir and Freundlich Isotherm Models

Adsorption isotherms commonly define the relation between the adsorbed amount of the DF (solute) onto the surface of the P-G-ILO nanohybrid (solid) and what remains in the bulk liquid phase at constant temperature. The adsorption data were equipped in the nonlinear equation of the Langmuir and Freundlich isotherm model to evaluate the adsorption capacity (*q*_m_) and nature of interaction of DF molecules with the P-G-ILO nanohybrid surface. The adsorption capacity of ILO was also calculated. The nonlinear forms of the Langmuir [29] and Freundlich [30] isotherm equation are given as follows (Equations (6) and (7)).

(6) $$q_{e}=\frac{q_{m}bC_{e}}{\left(1+bC_{e}\right)}$$

where *q*_m_ represents the maximum monolayer adsorption capacity in mg/g of the P-G-ILO nanohybrids and *b* represents the free energy adsorption (Langmuir constant) in L/mg.

**Figure 5:**
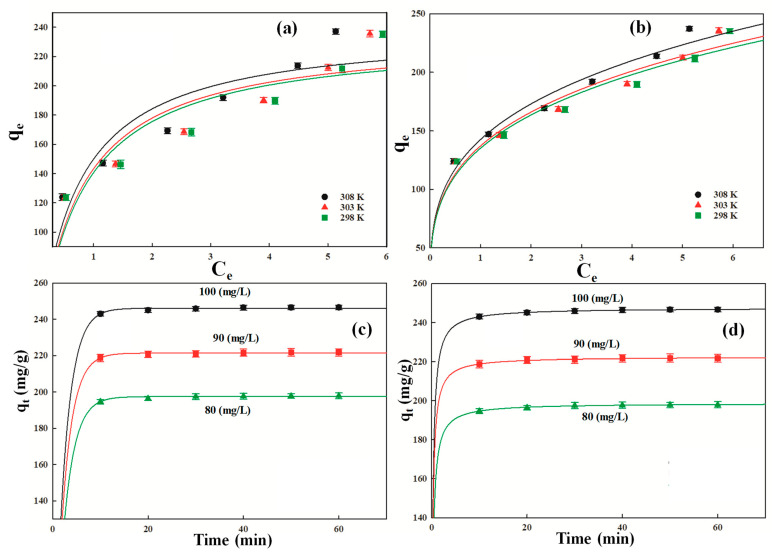
(**a**) Langmuir isotherm, (**b**) Freundlich isotherm, (**c**) PFO kinetic model, and (**d**) PSO kinetic model plots of the DF adsorption over P-G-ILO nanohybrids.

The Langmuir isotherm model pronounces that the adsorption of DF molecules occurs at specific binding sites of the P-G-ILO nanohybrid surface, which are energetically the same without any interaction between adjacent adsorbed (DF) molecules. The *q*_m_ values were assessed from the plot of *C*_e_ vs. *q*_e_ (Figure 5a) and found to be 254, 256, and 258 mg/g at 298, 303, and 308 K, respectively (Table 3). The *q*_m_ values slightly increase with increasing temperature, which supported the endothermic nature of adsorption of DF onto the P-G-ILO nanohybrid surface [31]. The *q*_e_ of the ILO nanoparticle was also investigated under similar conditions, and the calculated values were 233, 234, and 239 mg/g at 298, 303, and 308 K, respectively. The isotherm plots (Figure S3a,b) and calculated parameters (Table S2) are given in the supplementary data. The *q*_m_ values were compared with several other adsorbents (Table 4) [32,33,34,35,36,37,38]. The value of free energy of adsorption varies between 1.70, 1.78, and 2.05 (L/mg). The magnitude of free energy of adsorption increases with the increase in the temperature, which suggested strong binding of DF molecules at the higher temperature onto the P-G-ILO nanohybrid surface [39]. In order to inspect whether the adsorption of DF was favorable or not, the separation factor (*R*_L_) was calculated by *R*_L_ ($=\frac{1}{1+bC_{0}}$). The calculated values of *R*_L_ were 0.0058, 0.0055, and 0.0048 at 298, 303, and 308 K, respectively. The *R*_L_ values greater than zero and lower than one (0 < *R*_L_ < 1) recommended the favorable adsorption of DF, and the lowest *R*_L_ value was observed at a higher temperature, which suggested more favorable DF adsorption at high temperatures. The correlation coefficient (*R*^2^) values and standard error of estimate (*SEE*) were evaluated to be 0.928, 0.948, and 977 and 12.84, 10.95, and 7.25 at 298, 303, and 308 K, respectively.

(7)  qe=KfCe1n

where *K_f_* (mg/g)(L/mg)^1/*n*^ = Freundlich adsorption capacity 1/*n* = P-G-ILO nanohybrids surface heterogeneity 

The Freundlich adsorption isotherm confirmed that the single site onto the P-G-ILO nanohybrid surface may hold more than one DF molecule, forming multilayers onto the heterogeneous adsorbent surface (Figure 5b). The calculated values of *K_f_* (mg/g) (L/mg)^1/*n*^ and 1/*n* were 153, 161, and 165 (mg/g) (L/mg)^1/*n*^ and 0.289, 0.285, and 0.268 at 298, 303, and 308 K, respectively. The 1/*n* values exist in the range of 0–1, specifying favorable adsorption of DF onto the P-G-ILO nanohybrid surface at all the three temperatures (298, 303, 308 K) [40]. The correlation coefficient (*R*^2^) values and standard error of estimate (*SEE*) were estimated to be 0.982, 0.991, 0.981 and 6.30, 4.42, 6.52 at 298, 303, and 308 K, respectively (Table 5).

The higher values of *R*^2^ and lower values of *SEE* for the Freundlich isotherm stipulated that adsorption data of DF adsorption are the best fitted to Freundlich isotherm. Therefore, the adsorption of DF occurs through the multilayer formation onto the heterogeneous surface of P-G-ILO nanohybrids.

### 3.6. Nonlinear PFO Kinetic Models

The adsorption rate of CF was extracted through fitting the adsorption data to the nonlinear PFO kinetic equation [41] and PSO kinetic equation [42] (Equations (8) and (9)). The PFO kinetic models explain the rate of adsorbate sequestration on ion exchange adsorbents, and the PSO kinetic models suggested that adsorbates hold onto two active sites of adsorbents [43].

(8) $$q_{t}=q_{e}\left(1-e^{-k_{1}\cdot t}\right)$$

(9) $$q_{t}=\frac{\left(k_{2}q_{e}^{2}\cdot t\right)}{\left(1+k_{2}q_{e}\cdot t\right)}$$

The *k*_1_ (1/min) (pseudo first order rate constant), *k*_2_ (PSO rate constant), *q*_e_ (adsorption capacity), *R*_2_, and *SEE* were determined from *q*_t_ versus *t* plots (Figure 5c,d). The *k*_1_ values were 0.42, 0.44, and 0.43 (1/min), respectively, for the 80, 90, and 100 mg/L of DF concentrations. The values of *R*^2^ for the pseudo first order (0.822, 0.882, and 0.842 for the 80, 90, and 100 mg/L DF solutions, respectively) were lower than for the pseudo second order (0.988, 0.982, and 0.996 for the 90 and 100 mg/L DF solutions, respectively). The *SEE* values of pseudo first order (0.59, 0.44, and 0.62) were higher than the pseudo second order (0.153, 1.70, and 0.092) (Table 6). The above observation authenticated that the adsorption of DF was directed by the PSO kinetic models. 

### 3.7. Adsorption Thermodynamic of DF Removal

Thermodynamic study provides the information about the energetic change associated with DF removal from the wastewater. Figure S3c displays the van’t Hoff plot (Equation (10)), which gives the value of the entropy and enthalpy change during the adsorption of the DF over the P-G-ILO nanohybrid. The overall change in the free energy (ΔG°) was calculated with the help of the Gibbs equation (Equation (11)).

(10) $$log\left(\frac{q_{e}}{C_{e}}\right)=\frac{\Delta S{}^{\circ}}{2.303R}-\frac{\Delta H{}^{\circ}}{2.303RT}$$

(11) ΔG°=ΔH°−TΔS°

The positive values of entropy change (∆*S*°) (0.041–0.107 kJ/mol K) and changes in enthalpy (∆*H*°) (2.1–14.2 kJ/mol) signify increased randomness onto the P-G-ILO nanohybrids surface, and adsorption of DF was endothermic in nature [44,45]. The negative value of the free energy change (ΔG°) (10.2–12.0 kJ/mol) demonstrated that sequestration of DF onto the P-G-ILO nanohybrid was thermodynamically spontaneous in nature (Table 7).

### 3.8. Regeneration and Reusability of the P-G-ILO Nanohybrid

The discarding of the unexploited adsorbent using the traditional method was not correct; for example, hazardous waste may leach from the spent adsorbent landfilling, while burning may release the harmful gases [46]. Therefore, the regeneration of the spent adsorbent is interestingly pleasing and adds to the adsorbent’s reusability. The experiment for the regeneration of P-G-ILO nanohybrid was accomplished by shaking the 100 mg of spent P-G-ILO nanohybrid with 25 mL of 0.1 M NaOH solution for 60 min. The regenerated P-G-ILO nanohybrid was carefully washed with double distilled water to confiscate any traces of NaOH and used again for adsorption experiments. The adsorption-desorption experiment was repeated for five consecutive cycles. The P-G-ILO nanohybrid retained its adsorption efficiency largely even after five adsorption/desorption cycles sequestering 85% of DF, while the desorption efficiency was 83% (Figure S3d). The admirable reusability of the P-G-ILO nanohybrid up to five adsorption-desorption cycles established its cost effectiveness.

## 4. Conclusions

The polyacrylamide/Gelatin-FeLaO_3_ nanocomposite (P-G-ILO nanohybrid) was synthesized and characterized. The XRD spectra of ILO nanoparticles suggested that nanoparticles were purely crystalline in nature, however, P-G-ILO nanohybrid spectra were found to be crystalline in nature. The surface area was calculated to be 2.43 m^2^/g for the ILO and 7.5 m^2^/g for the P-G-ILO nanohybrid. The average particle size of the P-G-ILO nanohybrid was 50–55 nm. Response surface methodology was used for the statistical optimization of diclofenac adsorption from the wastewater. The adsorption capacity (*q*_e,_ mg/g) of P-G-ILO nanohybrid was larger (254, 256, and 258 mg/g) than the ILO nanoparticle (239, 234, and 233 mg/g). The Freundlich isotherm model was the best fitted, as it gives the higher values of correlation coefficient (R^2^ = 0.982, 0.991 and 0.981) and lower value of standard error of estimate (*SEE* = 6.30, 4.42 and 6.52), which suggested multilayered adsorption of DF. The PSO kinetic models directed the adsorption of the DF, which represents the adsorption was endothermic and spontaneous in nature and randomness onto the P-G-ILO nanohybrid surface increases after the DF adsorption. The mechanism of adsorption of DF demonstrated that the adsorption of DF was mainly due to the electrostatic interaction, hydrogen bonding, and dipole interaction. The P-G-ILO nanohybrid was reusable for up to five adsorption/desorption cycles. The P-G-ILO nanohybrid has proven as an efficient, economic, and environmentally friendly material for the removal of DF from wastewater. 

## Figures and Tables

**Table 1:** Analysis of variance.

Variables	Unit	Factor	Range		
			−1	0	+1
Concentration	(mg/L)	A	50	75	100
pH		B	2	6	10
Time	(min)	C	10	35	60
Dose	(g/L)	D	0.4	0.9	1.4

**Table 2:** Fitting summary for the DF adsorption.

Source	Adjusted Standard Dev.	Adjusted R-Squared	Predicted R-Squared	R-Squared	PRESS	Column4
Linear	22.608	0.864	0.842	0.782	20,471.64	
2FI	16.83	0.942	0.912	0.850	14,024.17	
Quadratic	6.169	0.993	0.988	0.961	3594.69	Suggested
Cubic	2.56	0.999	0.997	0.879	11,353.64	Aliased

**Table 3:** Analysis of variance.

	Sum of		Mean	F	*p*-Value	
Source	Squares	df	Square	Value	Prob > F	
Model	93,485.03	14	6677.50	175.46	<0.0001	significant
A—Concentration	20,115.36	1	20,115.36	528.56	<0.0001	
B—pH	3289.14	1	3289.14	86.43	<0.0001	
C—Time	7.92	1	7.92	0.21	0.6548	
D—Dose	57,864.89	1	57,864.89	1520.48	<0.0001	
AB	379.96	1	379.96	9.98	0.0065	
AC	30.21	1	30.21	0.79	0.3870	
AD	5689.98	1	5689.98	149.51	<0.0001	
BC	7.73	1	7.73	0.20	0.6586	
BD	1275.51	1	1275.51	33.52	<0.0001	
CD	9.98	1	9.98	0.26	0.6161	
A^2^	0.068	1	0.068	1.774 × 10^−3^	0.9670	
B^2^	421.87	1	421.87	11.09	0.0046	
C^2^	32.34	1	32.34	0.85	0.3712	
D^2^	3296.04	1	3296.04	86.61	<0.0001	
Residual	570.85	15	38.06			
Lack of Fit	570.85	10	57.09			
Pure Error	0.000	5	0.000			
Cor Total	94,055.88	29				

**Table 4:** Comparison of *q*_m_ of various adsorbents for DF removal.

Adsorbent	*q*_m_	Isotherm/Kinetics	References
Chitosan-modified waste tire crumb rubber	70 mg/g	Freundlich/pseudo second	[32]
Chloride-modified zeolite	31 mg/g	----	[33]
Hexadecyltrimethylammonium bromide-modified zeolite	43 mg/g	----	[33]
Granular carbon nanotubes/alumina	106.5 μmol/g	Langmuir	[34]
Tea waste-derived activated carbon	91	Langmuir/pseudo second	[35]
Commercial activated carbon	76.98 mg/g	Freundlich/pseudo second	[36]
Reduced graphene oxide	59.67 mg/g	Liu isotherm	[37]
ZnFe_2_O_4_/chitosan magnetic	10 mg/g	BET multilayer model/pseudo second	[38]

**Table 5:** Isotherm parameters.

Temperature (K)	Langmuir					Freundlich			
	Parameters					Parameters			
	*q*_m_ (mg/g)	B (L/mg)	*R*_L_	*R* ^2^	*SEE*	*K_f_* (mg/g)(L/mg)^1/*n*^	1/n	*R* ^2^	*SEE*
308	258	2.05	0.0048	0.977	7.25	165	0.268	0.981	6.52
303	256	1.78	0.0055	0.948	10.95	161	0.285	0.991	4.42
298	254	1.70	0.0058	0.928	12.84	153	0.289	0.982	6.30

**Table 6:** Kinetic parameters.

Conc (mg/L)	*k*_1_ (1/min)	Pseudo First Order			Pseudo Second Order			
		*q*_e_ (cal) (mg/g)	*SEE*	*R* ^2^	k_2_ × 10^−2^ (g/mg min^−1^)	*q*_e_ (cal) (mg/g)	*SEE*	*R* ^2^
80	0.42	197	0.59	0.822	2.4	198	0.153	0.988
90	0.44	221	0.44	0.882	2.6	222	0.170	0.982
100	0.43	246	0.62	0.842	2.1	247	0.092	0.996

**Table 7:** Thermodynamic parameters.

Adsorbate	Conc (mg/L)	∆*H*° (kJ/mol)	∆*S*° (kJ/mol K)	−∆*G*° (kJ/mol)		
				298 K	303 K	308 K
DCF	80	14.2	0.085	11.2	11.6	12.0
	90	21.8	0.107	10.2	10.7	11.3
	100	2.1	0.041	10.2	10.4	10.6

## Data Availability

The data presented in this study are available on request from the corresponding author.

## Supplementary Materials

The following supporting information can be downloaded at: https://www.mdpi.com/article/10.3390/nano13212835/s1. Figure S1. SEM images of the DF-P-G-ILO nanohybrids. Figure S2. The zero point charge determination of P-G-ILO nanohybrids. Figure S3. (a) Langmuir, (b) Freundlich isotherm plot of ILO nanoparticles, (c) thermodynamic plot for the DF adsorption onto P-G-ILO nanohybrids, and (d) Adsorption/desorption curve of P-G-ILO nanohybrids. Table S1: actual and predicted values of adsorption capacities. Table S2: Isotherm parameters of FLO.
