# Supplementary material for: Emerging Mesoporous Polyacrylamide/Gelatin–Iron Lanthanum Oxide Nanohybrids towards the Antibiotic Drugs Removal from the Wastewater
Source: Nanomaterials (Basel). 2023 Oct 26;13(21):2835. doi: 10.3390/nano13212835 (PMC10649728; doi:10.3390/nano13212835)
Supplement: Supplementary file 1 [file nanomaterials-13-02835-s001.zip › nanomaterials-2646467-supplementary.pdf]

Supplementary Materials

Emerging Mesoporous Polyacrylamide/Gelatin–Iron Lanthanum Oxide Nanohybrids towards the Antibiotic Drugs Removal from the Wastewater

Nazish Parveen [1,*], Fatimah Othman Alqahtani [1], Ghayah M. Alsulaim [1], Shada A. Alsharif [2], Kholoud M. Alnahdi [3], Hasna Abdullah Alali [4], Mohamad M. Ahmad [4,5] and Sajid Ali Ansari [4,*]

1 Department of Chemistry, College of Science, King Faisal University, Al-Ahsa, P.O. Box 380, Hofuf 31982, Saudi Arabia
2 University College of Umlij, University of Tabuk, Tabuk 71491, Saudi Arabia
3 Department of Physics, Faculty of Science, University of Tabuk, Tabuk 71491, Saudi Arabia
4 Department of Physics, College of Science, King Faisal University, Al-Ahsa, P.O. Box 400, Hofuf 31982, Saudi Arabia
5 Department of Physics, Faculty of Science, The New Valley University, El-Kharga 72511, Egypt
* Correspondence: nislam@kfu.edu.sa (N.P.); sansari@kfu.edu.sa (S.A.A.); Tel.: +966-13-589-6915 (N.P.)

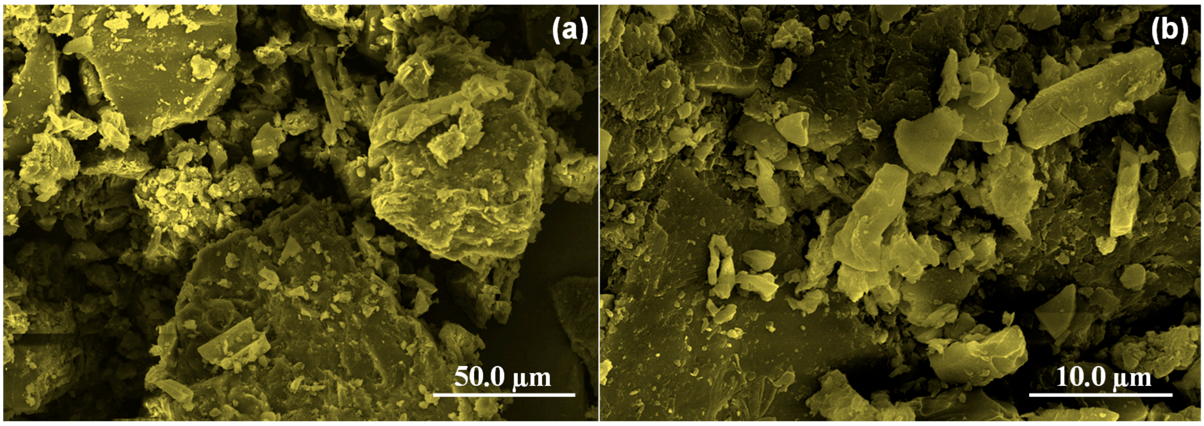

Figure S1. SEM images of the DF-P-G-ILO nanohybrids.

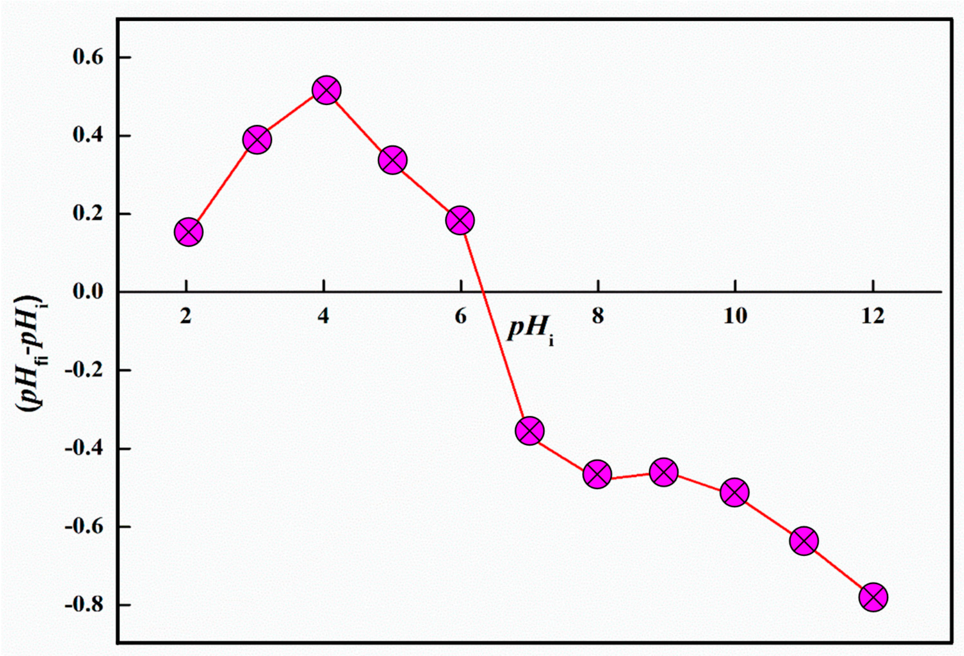

Figure S2. The zero point charge determination of P-G-ILO nanohybrids.

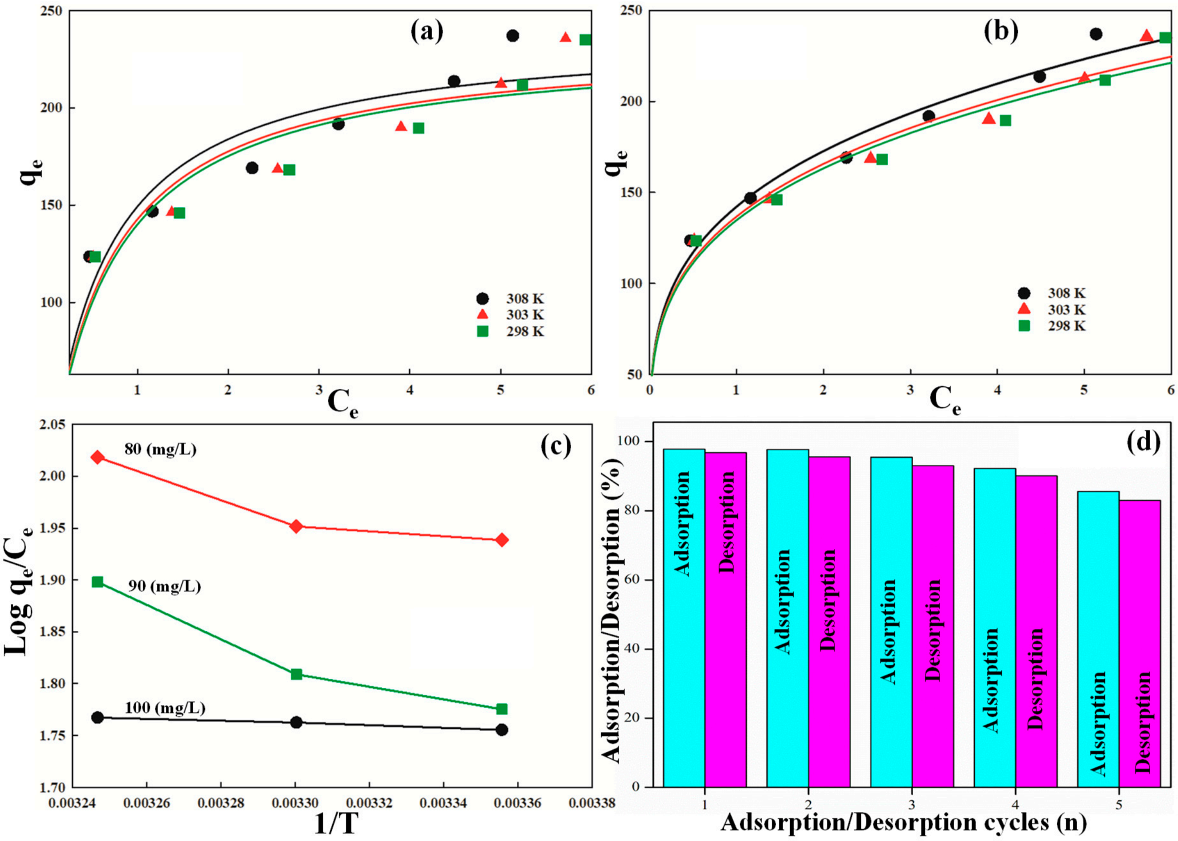

Figure S3. (**a**) Langmuir, (**b**) Freundlich isotherm plot of ILO nanoparticles, (**c**) thermodynamic plot for the DF adsorption onto P-G-ILO nanohybrids, and (**d**) Adsorption/desorption curve of P-G-ILO nanohybrids.

Table S1: actual and predicted values of adsorption capacities

Run	Concentration (mg/L)	pH	Time (min)	Dose (g/L)	Actual Value q_e	Predicted Value q_e
1	75	6	10	0.9	77.72	78.13
2	75	6	35	0.9	82.45	82.33
3	75	6	35	0.9	82.45	82.33
4	50	2	10	0.4	119.13	124.14
5	100	10	10	1.4	55.1	48.01
6	50	10	60	0.4	86.41	89.15
7	100	10	60	0.4	187.49	186.73
8	75	6	35	1.4	53.14	61.3
9	75	6	60	0.9	79.62	79.46
10	50	10	10	1.4	26.45	31.36
11	50	6	35	0.9	55.15	48.74
12	100	2	10	1.4	67.72	68.32
13	75	6	35	0.9	82.45	82.33
14	100	10	60	1.4	53.55	51.89
15	50	2	60	1.4	33.27	27.79
16	50	10	60	1.4	27.34	29.75
17	100	2	60	0.4	241.54	239.98
18	75	6	35	0.9	82.45	82.33
19	100	6	35	0.9	108.93	115.6
20	75	10	35	0.9	61.24	56.05
21	75	6	35	0.9	82.45	82.33
22	75	6	35	0.4	182.6	174.7
23	75	6	35	0.9	82.45	82.33
24	75	2	35	0.9	77.65	83.09
25	100	2	10	0.4	241.54	235.72
26	100	2	60	1.4	68.64	69.43
27	100	10	10	0.4	170.86	179.69
28	50	2	10	1.4	34.84	32.18
29	50	10	10	0.4	91.81	87.61
30	50	2	60	0.4	119.24	122.91

Table S2: Isotherm parameters of FLO

	Langmuir					Freundlich			
**Temperature (K)**	**Parameters**					**Parameters**			
	***q_m* (mg/g)**	**b (L/mg)**	***R_L***	***R^2***	***SEE***	***K_f* (mg/g)(L/mg)$^{1/n}$**	**1/n**	***R^2***	***SEE***
308	239	1.67	0.0059	0.833	19.27	142	0.28	0.960	9.38
303	234	1.56	0.0063	0.807	20.75	137	0.27	0.946	10.77
298	233	1.50	0.0066	0.800	20.72	135	0.27	0.942	11.11
